# Somatic cell count in bovine quarter milk samples culture positive for various *Staphylococcus* species

**DOI:** 10.1186/s13028-022-00649-8

**Published:** 2022-11-26

**Authors:** Suvi Taponen, Vesa Myllys, Satu Pyörälä

**Affiliations:** 1 Department of Production Animal Medicine, Faculty of Veterinary Medicine, University of Helsinki, Paroninkuja 20, 04920 Saarentaus, Finland; 2 Vetcare Oy, Liedontie 45, 04600 Mäntsälä, Finland

**Keywords:** Bovine mastitis, Milk somatic cell count, NAS, Non-aureus staphylococci, SCC, *Staphylococcus*

## Abstract

**Background:**

Non-aureus staphylococci (NAS) are the most prevalent group of bacteria isolated in bovine mastitis milk in Finland and many other countries. They usually cause subclinical or mild clinical mastitis. The increase in milk somatic cell count (SCC) during NAS intramammary infection varies from slight to marked, reflecting the severity of infection in the quarter. Limited evidence has indicated that NAS species may have different impact on milk SCC. We used a large data set originating from a prevalence study, including isolates from quarter milk samples and the SCCs of the respective quarters, to study the effect of different NAS species on quarter milk SCC.

**Results:**

Staphylococcal species of a total of 1265 isolates, originally identified as NAS, were analysed with MALDI-TOF MS. The most prevalent NAS species were *S. epidermidis*, *S. simulans*, *S. chromogenes* and *S. haemolyticus*. Forty-two isolates appeared to be *S. aureus.* Geometric mean milk SCC of all quarter samples was 114,000 cells/mL and median 126,000 cells/mL. *Staphylococcus* species had a significant effect on the SCC of the quarter. The highest SCCs were caused by *S. aureus, S. agnetis/S. hyicus* (these two species cannot be distinguished with MALDI-TOF MS) and *S. simulans.* The mean SCCs of milk samples that were culture positive for these three species did not differ significantly from each other but were significantly higher than the mean SCCs of milk samples positive for any other species. The mean SCC of milk samples positive for *S. chromogenes* was significantly higher than those of milk samples positive for *S. epidermidis* or *S. warneri*.

**Conclusion:**

Our results confirm that different *Staphylococcus* species have different impacts on milk SCC, as shown in previous studies. *S. aureus* caused the highest SCC, as expected, but the SCCs caused by *S. agnetis/S. hyicus* and *S. simulans* did not differ significantly from that of *S. aureus*. Other *Staphylococcus* species may also cause high SCC but are often isolated also from quarters with SCC on the level of healthy quarters.

## Background

Non-aureus staphylococci (NAS) are the most prevalent group of bacteria isolated in bovine mastitis milk in Finland [1, 2] and many other countries [3]. Mastitis caused by NAS is usually subclinical or mildly clinical. NAS intramammary infection (IMI) results in inflammatory reaction in the quarter, which is reflected as an increased milk somatic cell count (SCC) and possibly decreased milk production in the cow [4, 5]. The rise of SCC in NAS intramammary infection varies from a slight to a marked increase [3]. The immune reaction of the infected udder quarter and the following inflammation and increased milk SCC are influenced by the characteristics of both the cow and the microbe. Non-aureus *Staphylococcus* species and strains of species have been found to have different impacts on milk SCC. Supré et al. [6] studied NAS mastitis in three Belgian dairy herds and found *S. chromogenes, S. simulans*, and *S. xylosus* to induce an increase in the SCC that could even be comparable with that of *S. aureus*. Experimental mastitis studies in which quarters of cows were challenged with different NAS species or strains have shown differences in the intensity of the inflammatory reaction of the udder quarter between NAS species or strains [5, 7]. The inflammatory reactions shown in these studies are not comparable with natural NAS IMI because of high infection doses but reflect the possible differences in immunity effects between NAS species and strains. Simojoki et al. [5] found quarters challenged with *S. simulans* to have a more intense immune reaction measured by several markers of inflammation, including SCC, than quarters challenged with *S. epidermidis*. Piccart et al. [7] challenged bovine quarters with *S. fleurettii* and two strains of *S. chromogenes*, one isolated in mastitic milk and the other from teat apex. A *S. chromogenes* strain originating from bovine mastitis tended to increase milk SCC more than *S. chromogenes* strain originating from teat apex, or *S. fleurettii* originating from bovine mastitis. Our aim was to use a large data set collected during a mastitis prevalence study [8] to study the effect of NAS species isolated in bovine udder quarters on quarter milk somatic cell count of the same quarters.

## Methods

The NAS isolates used in this study were collected during a mastitis prevalence study in 1995 [8]. The sampling is described in detail in Myllys et al. [8]. Briefly, all quarters of all lactating cows in 238 private dairy herds selected randomly from all dairy herds in Finland were sampled by dairy advisors. In total, 10 410 quarter milk samples from 2 648 cows from 238 herds were collected. Samples, one for microbial culturing and another for SCC count, were collected using aseptic technique, cooled, and sent in cooler bags with cold packs to the laboratory of the National Veterinary and Food Research Institute in Helsinki. Samples were cultured on Trypticase Soy Agar plates containing 5% blood, and bacterial species were identified using routine bacteriological methods [9, 10]. Quarter milk SCC was determined with an electric counter (Fossomatic Milk Analysis, Foss Electric, Hillerød, Denmark).

In the present study we used 1162 isolates identified as NAS in the study by Myllys et al. [8], and the SCC results of these isolates. The isolates stored at − 80 °C were thawed and cultured on bovine blood agar plates (TammerBiolab Oy, Tampere, Finland). Despite the long storage, almost all samples yielded bacterial growth. The purity of the bacterial growth was controlled. The *Staphylococcus* species of the pure cultures were analysed with Matrix Assisted Laser Desorption/Ionization Time of Flight Mass Spectrometry (MALDI-TOF MS, Microflex LT, Bruker Daltonic Gmbh, Bremen, Germany) using the direct transfer protocol [11]. For species identification, the Bruker database and an in-house database containing some NAS species not included in the Bruker database were used. The in-house NAS database included the following NAS strains: *S. agnetis* DSM 23656, *S. agnetis* DSM 23657, *S. agnetis* DSM 23658, *S. devriesei* DSM 25293, *S. rostri* DSM 21968, and *S. rostri* DSM 21969. Som NAS species have been recently reclassified as mammaliicoccal species [12]. This applies in our study to *S. sciuri* which is now *Mammaliicoccus (M.) sciuri*. Isolates identified as *S. haemolyticus* may include some isolates of a novel species *S. borealis* as MALDI-TOF MS does not differentiate them [13].

In total, 1162 isolates, originally identified as NAS, were analysed with MALDI-TOF MS. Instead of the commonly used score value ≥ 2.000 for reliable species identification, we used the score value ≥ 1.700, which has shown to improve the performance of MALDI-TOF MS when classifying NAS isolates [11]. However, only 16% of the isolates had a score value < 2.000, and at least two of the three repeats gave the same identification. Isolates not reliably identified with MALDI-TOF MS (score value < 1.7) or identified to belong to other microbial genera than *Staphylococcus* were excluded from further analyses. The final data consisted of 1055 *Staphylococcus* isolates from 207 dairy herds. The number of samples per herd varied from 1 to 39. The mean and median numbers of samples per herd were 5.23 and 4, respectively. Because MALDI-TOF MS cannot distinguish between the closely related species *S. agnetis* and *S. hyicus*, these two species were grouped as *S. agnetis/S. hyicus*.

Samples in which more than one *Staphylococcus* species were isolated were excluded from the study. For statistical analyses, *Staphylococcus* species with ≤ 10 isolates were grouped as “other *Staphylococcus* species”. For graphic illustration of distribution of SCC from milk samples with different *Staphylococcus* species, the SCC was grouped in 8 groups: 1: 1–50,000 cells/mL, 2: 51,000–100,000 cells/mL, 3: 101,000–200,000 cells/mL, 4: 201,000–300,000 cells/mL, 5: 301,000–400,000 cells/mL, 6: 401,000–500,000 cells/mL, 7: 501,000–1,000,000 cells/mL, and 8: > 1,000,000 cells/mL. The class 1 was determined as non-mastitic [14]. Geometric means were calculated separately for all samples and mastitic samples (classes 2–8).

The effect of *Staphylococcus* species on the natural log-transformed SCC of the quarter milk sample was studied using General Linear Model, in which lnSCC was the outcome variable, *Staphylococcus* species the explaining variable, and herd was included as a random factor. The effect of *Staphylococcus* species was calculated separately for the group of all samples and for the group of mastitic samples. A P-value < 0.05 was considered significant. The Bonferroni post hoc test was used to find the statistical differences between each species. The statistical analyses were performed using IBM SPSS Statistics version 26.

## Results

The *Staphylococcus* isolates belonged to the following 21 species (in order of prevalence): *S. epidermidis* (279), *S. simulans* (225), *S. chromogenes* (158), *S. haemolyticus* (114), *S. warneri* (105), *S. xylosus* (43), *S. aureus* (42), *S. agnetis/S. hyicus* (25), *S. cohnii* (24), *S. capitis* (10), *S. devriesei* (6), *M. sciuri* (5), *S. equorum* (4), *S. pasteuri* (4), *S. hominis* (2), *S. lugdunensis* (2), *S. nepalensis* (2), *S. saprophyticus* (2), *S. arlettae* (1), *S. rostri* (1), and *S. succinus* (1). The 42 isolates identified as *S. aureus* were identified originally as coagulase-negative staphylococci by Myllys et al. [9]. In that study, 362 isolates were identified as *S. aureus*.

The geometric mean milk SCC of all quarter samples culture positive for NAS was 114,000 cells/mL. The median was 126,000 cells/mL and the interquartile range (IQR) 38,000 to 355,000 cells/mL. *Staphylococcus* species had a significant effect (P < 0.001) on the SCC of the quarter milk sample. The highest SCCs were caused by *S. aureus, S. agnetis/S. hyicus* and *S. simulans* (Table 1). The geometric mean SCCs of following species differed statistically significantly: *S. aureus* from *S. epidermidis, S. chromogens, S. haemolyticus, S. warneri*, and *S. xylosus, S. agnetis/S. hyicus* from *S. epidermidis, S. warneri* and *S. xylosus, S. simulans* from *S. epidermidis, S. haemolyticus, S, warneri* and *S. xylosus*, *S. chromogenes* from *S. warneri*, and *S. haemolyticus* from *S. warneri*.

**Table 1:** The geometric mean somatic cell counts (SCCs) and 95% confidence intervals of all milk samples and milk samples classified as mastitic*, culture positive for different *Staphylococcus* species, and the percentages of non-mastitic quarter milk samples in each *Staphylococcus* species

*Staphylococcus* species isolated	N	Geomean SCC × 1000 all quarters	95% CI × 1000	Geomean SCC × 1000 mastitic quarters	95% CL × 1000	% Non-mastitic quarters
*S. agnetis/S. hyicus*	25	342	182–642	472	299–744	12.0
*S. aureus*	42	327	201–532	529	370–755	14.3
*S. chromogenes*	158	131	102–168	175	146–210	13.9
*S. cohnii*	24	104	55–198	279	163–476	33.3
*S. epidermidis*	279	80	66–97	282	238–333	41.9
*S. haemolyticus*	114	111	83–149	295	213–377	33.3
*S. simulans*	225	216	175–267	323	277–377	13.3
*S. warneri*	105	52	38–71	219	163–295	50.5
*S. xylosus*	43	81	50–131	175	117–260	32.6
Other species^a^	40	50	30–82	172	109–271	45.0

^*^SCC > 50,000 cells/mL

^a^Other species: 1 *S. arlettae*, 10 *S. capitis*, 6 *S. devriesei*, 4 *S. equorum*, 2 *S. hominis*, 2 *S. lugdunensis*, 2 *S. nepalensis*, 4 *S. pasteuri*, 1 *S. rostri*, 2 *S. saprophyticus*, 5 *M. sciuri*, 1 *S. succinus*

**Fig. 1:**
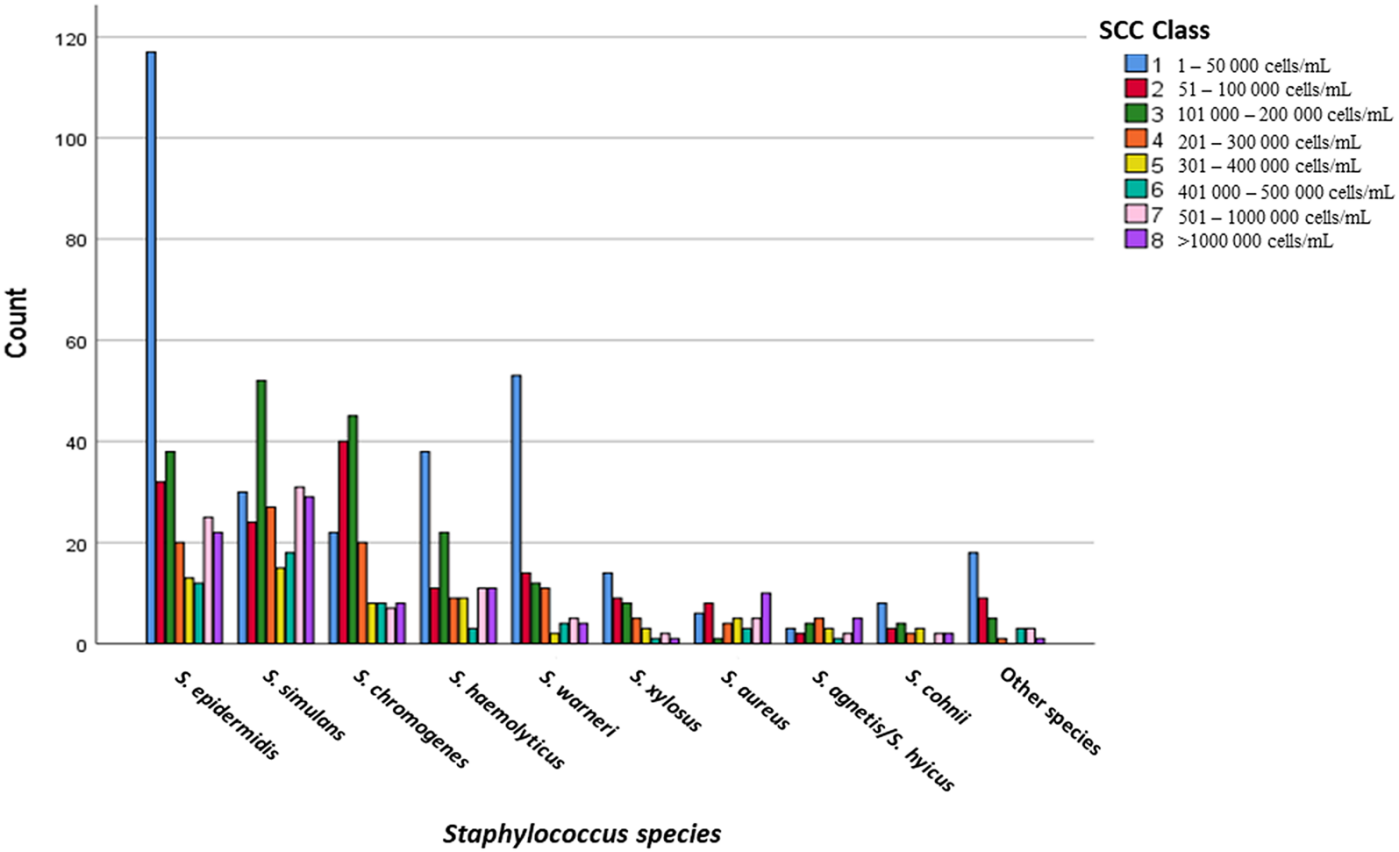
Distribution of milk somatic cell counts classes of milk samples culture positive with *Staphylococcus* species

In total 29.3% of all samples were classified as non-mastitic, but the proportion of non-mastitic samples varied greatly between the species (Table 1). The geometric mean SCC of non-mastitic samples was 15,000 cells/mL and that of mastitic samples 267,000 cells/mL. The median SCC of mastitic samples was 222,000 cells/mL and the IQR 113,000–490,000 cells/mL. Figure 1 illustrates the division of milk samples positive for different *Staphylococcus* species into different cell classes. The percentages of isolates in each cell class are shown in Table 2. The proportion of the lowest cell class 1 (≤ 50,000 cells/mL), classified as non-mastitic, is highest in samples positive for *S. warneri* (50.5%) and *S. epidermidis* (41.9%) (Table 1), whereas the proportion of class 8, with the highest cell count (> 1,000,000 cells/mL), is highest in samples positive for *S. aureus* (23.8%) and *S. agnetis/S. hyicus* (20.0%) (Table 2). The geometric mean SCC of mastitic samples was highest in samples positive for *S. aureus* and *S. agnetis/S. hyicus*, followed by *S. simulans, S. haemolyticus* and *S. epidermidis* (Table 1). Significant differences (P < 0.05) existed between the following species: *S. aureus* differed from *S. epidermidis*, *S. chromogenes*, *S. haemolyticus*, *S. warneri*, and *S. xylosus, S. agnetis/S. hyicus* differed from *S. chromogenes, S. warneri* and *S. xylosus*, and *S. simulans* differed from *S. chromogenes, S. warneri*, and *S. xylosus*.

**Table 2:** The percentages of isolates of different *Staphylococcus* species in different milk somatic cell count (SCC) classes

*Staphylococcus* species	% Isolates in different milk SCC classes							
	1	2	3	4	5	6	7	8
*S. agnetis/S. hyicus*	12.0	8.0	16.0	20.0	12.0	4.0	8.0	20.0
*S. aureus*	14.3	19.0	2.4	9.5	11.9	7.1	11.9	23.8
*S. chromogenes*	13.9	25.3	28.5	12.7	5.1	5.1	4.4	5.1
*S. cohnii*	33.3	12.5	16.7	8.3	12.5	0.0	8.3	8.3
*S. epidermidis*	41.9	11.5	13.6	7.2	4.7	4.3	9.0	7.9
*S. haemolyticus*	33.3	9.6	19.3	7.9	7.9	2.6	9.6	9.6
*S. simulans*	13.3	10.7	23.1	12.0	6.7	8.0	13.8	12.4
*S. warneri*	50.5	13.3	11.4	10.5	1.9	3.8	4.8	3.8
*S. xylosus*	32.6	20.9	18.6	11.6	7.0	2.3	4.7	2.3
Other species^a^	45.0	22.5	12.5	2.5	0.0	7.5	7.5	2.5

The SCC classes: 1: 1–50,000 cells/mL, 2: 51000–100,000 cells/mL, 3: 101,000–200,000 cells/mL, 4: 201,000–300,000 cells/mL, 5: 301,000–400,000 cells/mL, 6: 401,000–500,000 cells/mL, 7: 501,000–1,000,000 cells/mL, and 8: > 1,000,000 cells/mL

^a^Other species: 1 *S. arlettae*, 10 *S. capitis*, 6 *S. devriesei*, 4 *S. equorum*, 2 *S. hominis*, 2 *S. lugdunensis*, 2 *S. nepalensis*, 4 *S. pasteuri*, 1 *S. rostri*, 2 *S. saprophyticus*, 5 *M. sciuri*, 1 *S. succinus*

## Discussion

We found significant differences in quarter milk SCC from quarters culture positive for different *Staphylococcus* species commonly associated with bovine IMI. The well-known major pathogen *S. aureus* was associated with the highest milk SCC, although the *S. aureus* isolates in our data were originally incorrectly identified as NAS and may thus have some special characteristics compared to the correctly identified *S. aureus* population. The NAS species *S. agnetis/S. hyicus* and *S. simulans*, earlier associated with clinical mastitis or more intense inflammation [5, 15–17], were found in our study to cause milk SCC statistically not significantly different from that caused by *S. aureus*. These species, as well as *S. chromogenes*, were mainly (86–88%) isolated from mastitic quarters, whereas a large proportion (30–50%) of other species were isolated from non-mastitic quarters. For example, 42% of *S. epidermidis* and 33% *of S. haemolyticus* originated from non-mastitic quarters, which decreased the overall mean SCC calculated for these species. However, SCC in mastitic samples positive for these two species was on the same level with the SCC of *S. simulans* positive samples and did not differ significantly from the SCC of *S. agnetis/S. hyicus* or *S. simulans* positive samples. *Staphylococcus epidermidis* and *S. haemolyticus* were associated with high SCC also in the study by Nyman et al. [18]: the median SCC of milk samples from subclinical mastitis positive for *S. epidermidis* or *S. haemolyticus* was high and on the same level (from 1.1 to 2.0 million cells/mL) as the median SCCs of samples positive for *S. simulans* or *S. hyicus*. This may indicate that the species commonly isolated from healthy quarters mainly reside on teat skin or teat canal and lack effective virulence properties helping them to invade into the mammary gland. But once they manage to do so, they seem to cause an inflammatory reaction not very different from that of the NAS species predominantly associated with high SCC. Some strains may also be more virulent than other strains and one possible explanation is that the virulent strains cause IMI while the less virulent strains are controlled by the hosts’ immune defense already in the teat canal. Figure 1 shows that the biggest difference between the species is the proportion of isolates belonging to the SCC class 1 (non-mastitic). Distribution of isolates of different NAS species into the SCC classes illustrates better the effect of NAS species on SCC than the mean SCC. For example, 35.7% of *S. aureus*, 28.0% of *S. agnetis/S. hyicus* and 26.2% of *S. simulans* isolates belong to the two highest SCC classes, i.e., >500,000 cells/mL, while that proportion of all other species are < 20% (Table 2).

Supré et al. [6] followed quarters of 89 cows with diagnosed IMI on three herds with repeated sampling and found IMI by *S. chromogenes, S. simulans* and *S. xylosus* to increase milk SCC more than other NAS species isolated in quarter milk samples. The differences between SCC caused by these three NAS species and *S. aureus* infection were not statistically significantly different, although the actual geometric mean SCC of milk samples from *S. aureus* mastitis, 495,000 cells/mL, was higher than that of milk samples from *S. chromogenes* (226,000 cells/mL), *S. simulans* (130,000 cells/mL) or *S. xylosus* (85,000 cells/mL) mastitis. In contrast to the results of Supré et al. [6], *S. chromogenes* did not belong to the NAS species causing highest SCC in our study. Although most *S. chromogenes* were isolated from mastitic quarters, 80.4% were isolated from quarters with SCC ≤ 300,000 cells/mL (Table 2). Wuytack et al. [14] performed a cross-sectional sampling of approximately 25% of cows on eight herds. In this study, 39% of quarter milk samples originated from quarters with SCC ≤ 50,000 cells/mL and classified as non-mastitic. The NAS positive samples had a geometric mean SCC of 109,000 cells/mL, which is in agreement with the SCC from all samples in our study. The geometric mean of samples positive for *S. chromogenes* was 156,000 cells/mL and that of samples positive for *S. haemolyticus* 177,000 cells/mL [14]. A similar SCC level, 144,000 cells/mL, in quarters of newly calved primiparous cows infected with *S. chromogenes* was reported by Valckenier et al. [19]. Lower SCCs were reported by Condas et al. [20]. The geometric mean SCC of NAS-positive quarters was 70,000 cells/mL. *Staphylococcus agnetis*, *S. capitis*, *S. hyicus*, *S. gallinarum* and *S. simulans* were the species which increased the SCC most and the geometric mean SCC of these species was significantly higher than that of the whole NAS group. The geometric mean SCCs of samples positive for these species varied from 81,000 cells/mL for quarters positive for *S. agnetis* to 123,000 cells/mL for samples positive for *S. capitis*. For comparison, the mean SCC for *S. aureus* positive samples was 174,000 cells/mL. Condas et al. [20] used a large data set from a sampling similar to that of Wuytack et al. [14] and likely including a large proportion of isolates originating from non-mastitic quarters.

Sampling strategy has much effect on the results. Higher SCCs are obtained when samples are taken from subclinical or clinical mastitis cases, compared to studies where lactating quarters are sampled without knowledge about the inflammation status. This kind of sampling increases the probability that some isolates originate from the teat skin or teat canal of non-mastitic cows, as shown by 29% of isolates originating from non-mastitic quarters in our study and 39% in the study by Wuytack et al. [14]. Milk SCC varies a lot, from few thousands in a healthy quarter up to millions of cells/mL in a severely inflamed quarter. Mastitis is a dynamic phenomenon, and the infection status differs also during the IMI. Typically, the SCC is high at the beginning of a *Staphylococcus* IMI, but if the pathogen succeeds in avoiding the defensive reaction of the udder and the infection persists, the SCC usually decreases to a moderate but persistent level, with some variation [21].

In general, NAS cause subclinical or mild clinical mastitis, but differences between NAS species have been found in this respect. Some NAS species, in particular *S. agnetis/S. hyicus* and *S. simulans*, seem to be able to cause clinical mastitis more often than other NAS species [15–17, 20, 22]. In contrast, some NAS species, especially *S. epidermidis*, which is one of the most prevalent isolates from mastitis, has in experimental mastitis model shown to cause a milder inflammatory reaction than *S. simulans* [5]. However, *S. epidermidis* was associated with high SCC in milk samples originating from subclinical mastitis [18]. We found 42% of *S. epidermidis* isolates to originate from non-mastitic quarters but the SCC of mastitic *S. epidermidis* positive quarters not to be significantly different from the SCC caused by *S. agnetis/S. hyicus* or *S. simulans*. Unlike *S. chromogenes*, which typically affects heifers and primiparous cows and is considered part of bovine skin microbiota, *S. epidermidis* IMI is usually detected in multiparous cows [4, 18, 23]. *Staphylococcus epidermidis* is one of the most abundant bacterial colonisers of healthy human skin [24], but does it belong to bovine skin microbiota too? *S. epidermidis* has shown to be the NAS species commonly related to antimicrobial resistance and the species most commonly positive for the *mecA* gene encoding methicillin resistance [23, 25–27]. Persistent antimicrobial resistant *S. epidermidis* IMIs may cause a risk of emergence of antimicrobial-resistant staphylococci in the herd.

Following NAS species distribution in mastitis milk samples on herd level could help evaluation of transmission sources. For example, high presence of *S. epidermidis* may indicate transmission through milking equipment or possibly via milkers’ hands. Wuytack et al. [14] took swab samples from teat apices before and after milking. While most species were detected on teat skin both before and after milking, were *S. epidermidis* and *S. agnetis* detected only after milking, indicating infection from milking machine liners. However, strain typing is needed to ensure a common transmission source. In contrast, variation of different NAS species in milk samples indicates environmental transmission from manure, bedding, and other sources. At least 12 different NAS species have been isolated from bovine fecal samples [14], including species isolated frequently from milk samples. Laboratories using MALDI-TOF MS can already provide NAS speciation but this is not yet possible for laboratories that use commercial mastitis PCR tests. However, if the demand to add the most common NAS species in the tests’ selection of microbe species increases, the manufacturers of commercial mastitis PCR tests may become interested in providing such tests.

## Conclusion

Our results confirm that different *Staphylococcus* species affect milk SCC differently. As generally known, *S. aureus* caused the highest SCC, but the SCCs caused by *S. agnetis/S. hyicus* and *S. simulans* did not differ significantly from that of *S. aureus*. Other *Staphylococcus* species may also cause high SCC but are often isolated also from quarters with SCC on the level of healthy quarters.

## Data Availability

The datasets used and/or analysed during the current study are available from the corresponding author on reasonable request.
